# Hoveyda–Grubbs type metathesis catalyst immobilized on mesoporous molecular sieves MCM-41 and SBA-15

**DOI:** 10.3762/bjoc.7.4

**Published:** 2011-01-06

**Authors:** Hynek Balcar, Tushar Shinde, Naděžda Žilková, Zdeněk Bastl

**Affiliations:** 1 J. Heyrovský Institute of Physical Chemistry of AS CR, v.v.i, Dolejškova 3, 182 23 Prague 8, Czech Republic

**Keywords:** alkene metathesis, catalyst immobilization, hybrid catalysts, mesoporous molecular sieves, Ru–alkylidene complexes

## Abstract

A commercially available Hoveyda–Grubbs type catalyst (RC303 Zhannan Pharma) was immobilized on mesoporous molecular sieves MCM-41 and on SBA-15 by direct interaction with the sieve wall surface. The immobilized catalysts exhibited high activity and nearly 100% selectivity in several types of alkene metathesis reactions. Ru leaching was found to depend on the substrate and solvent used (the lowest leaching was found for ring-closing metathesis of 1,7-octadiene in cyclohexane – 0.04% of catalyst Ru content). Results of XPS, UV–vis and NMR spectroscopy showed that at least 76% of the Ru content was bound to the support surface non-covalently and could be removed from the catalyst by washing with THF.

## Introduction

**Figure 1:**
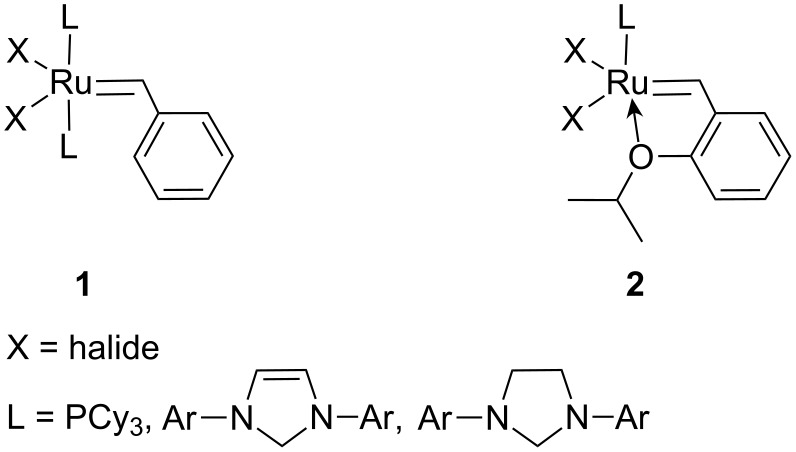
Grubbs **1** and Hoveyda–Grubbs **2** catalysts.

Ru–alkylidene complexes (Grubbs and Hoveyda–Grubbs catalysts, **1** and **2**, respectively, Figure 1) belong to the most active and frequently used metathesis catalysts. These catalysts are important tools in organic synthesis due to their high tolerance of heteroatoms in substrate molecules. Immobilization of these complexes on solid supports has attracted attention, because it opens the possibility for easy catalyst–product separation and catalyst reuse. The production of products free from catalyst residues is especially important in the synthesis of drugs and some other fine chemicals. Several strategies have been developed for the immobilization of Grubbs and Hoveyda–Grubbs catalysts on solid supports [1–6]. Generally, the complex can be attached to the supports: (a) by exchanging halide ligands X [7–11], (b) by exchanging phosphine and NHC ligands L [12–13], and (c) through the alkylidene ligand [14–19]. For these purposes, special ligand molecules with tags suitable for the reaction with the support surface (linkers) are used. This usually requires a sophisticated synthetic procedure. Moreover, the changes in the Ru coordination sphere may lead to the decrease in catalyst activity (e.g., the exchange of chloro ligands for carboxylates [10–11]).

Recently, a convenient method for the immobilization of Hoveyda–Grubbs catalysts was reported [20]. A second generation Hoveyda–Grubbs catalyst was immobilized on silica without any linkers by simply placing the Ru complex in contact with silica in a suspension. Heterogeneous catalysts (loading from 0.05 to 0.6 wt % Ru) were prepared, which were active in ring-opening metathesis polymerization (ROMP) of cyclooctene, ring-closing metathesis (RCM) of diallylsilanes and several types of cross-metathesis reactions. High stability of catalysts, reusability and low Ru leaching were also reported. However, the mode of attachment of the Ru species on the silica surface was unclear.

**Figure 2:**
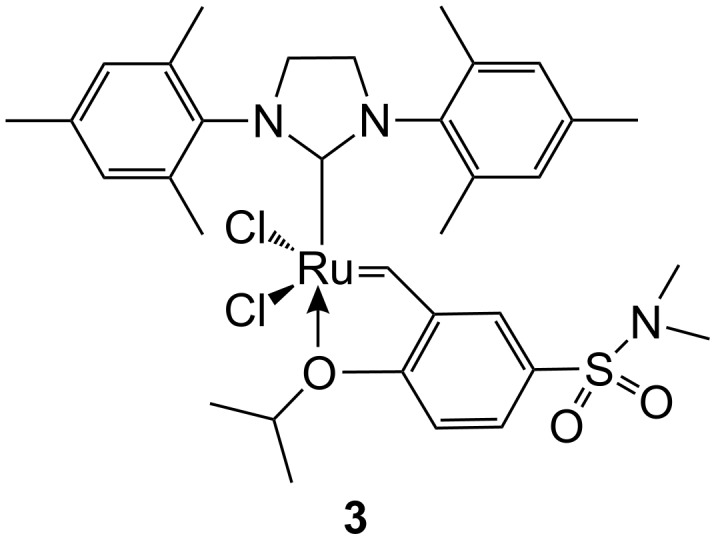
Zhan catalyst-1B.

The aims of this paper are the following: to report the immobilization of the Hoveyda–Grubbs type catalyst **3** (Figure 2, Zhan catalyst-1B) on mesoporous molecular sieves SBA-15 and MCM-41 as supports with this simple immobilization method; to describe the activity and stability of heterogeneous catalysts prepared; and to clarify the nature of the bond between the Ru complex and the support surface. Mesoporous molecular sieves are advanced siliceous materials [21], with high surface area, high pore volume and narrow distribution of pore size. Because of these unique qualities, they often find applications as supports of modern catalysts, including those used for metathesis reactions [22].

## Results and Discussion

### Catalyst activity

Mixing a toluene solution of **3** with dried MCM-41 and SBA-15, respectively, for 30 min at room temperature led to green solids, which after isolation and drying afforded heterogeneous catalysts **3**/MCM-41 and **3**/SBA-15. The immobilization proceeded almost quantitatively: 97% and 94% of the Ru complex was transferred from the solution onto the MCM-41 and SBA-15 surface, respectively. The established catalyst loading was 0.98 wt % and 0.93 wt %, for **3**/MCM-41 and **3**/SBA-15, respectively.

**Scheme 1:**
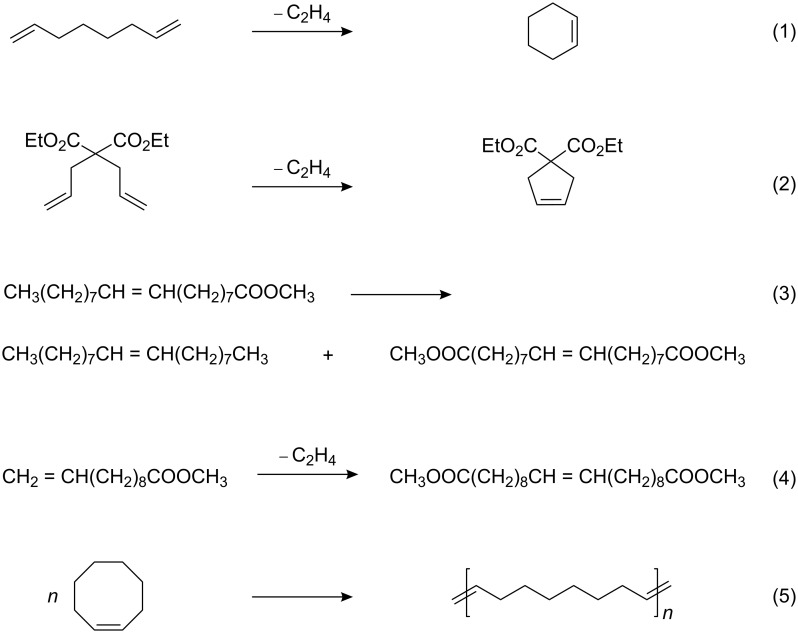
Metathesis reactions studied.

The catalytic activity was tested in the RCM of 1,7-octadiene (Scheme 1, entry 1) and diethyl diallylmalonate (DEDAM) (Scheme 1, entry 2), in the metathesis of methyl oleate (Scheme 1, entry 3) and methyl 10-undecenoate (Scheme 1, entry 4), and in the ROMP of cyclooctene (Scheme 1, entry 5).

**Figure 3:**
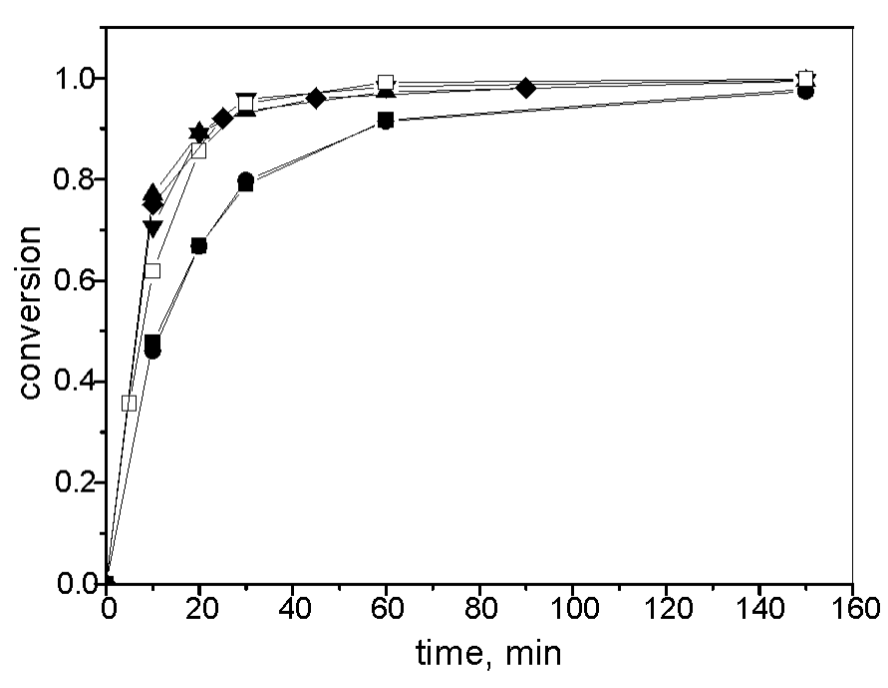
Conversion curves for the RCM of DEDAM with **3** in CH_2_Cl_2_ (open squares), **3**/SBA-15 in CH_2_Cl_2_ (inverted filled triangle), **3**/SBA-15 in benzene (filled diamond), **3**/SBA-15 in cyclohexane (filled triangle), and for the RCM of 1,7-octadiene with **3**/SBA-15 (filled squares) and **3**/MCM-41 (filled circles) in cyclohexane (*T* = 30 °C, molar ratio substrate/Ru = 600, c^0^_substrate_ = 0.2 mol/L).

Figure 3 shows conversion curves for the RCM of DEDAM in dichloromethane, benzene, and cyclohexane, and the RCM of 1,7-octadiene in cyclohexane. For the RCM of DEDAM, **3** (as a homogeneous catalyst) and **3**/SBA-15 were used. The reaction proceeded very rapidly in all solvents used (TOF at 10 min was approximately 2500 h^−1^). No decrease in the reaction rate was observed as a result of the immobilization of complex **3**. For the RCM of 1,7-octadiene, **3**/SBA-15 and **3**/MCM-41 in cyclohexane were used. The reaction profile was the same for both catalysts and the reaction rate was significantly lower compared to the RCM of DEDAM. The selectivity was near 100% in all cases (no side products were observed by GC).

The Ru leaching (in % of starting content of Ru in catalyst) for the reactions shown in Figure 3 is given in Table 1. It was observed that leaching was dependent on both the solvent and substrate used in the reaction – the highest leaching was found for the RCM of DEDAM in CH_2_Cl_2_ whilst negligible leaching was observed for the RCM of 1,7-octadiene in cyclohexane.

**Table 1:** Ru leaching for **3**/SBA-15.

Reaction	Solvent	Ru leaching	Maximal Ru content in product^a^

(1)	cyclohexane	0.04%	0.6 ppm
(2)	benzene	4%	28 ppm
(2)	cyclohexane	9%	66 ppm
(2)	dichloromethane	14%	100 ppm

molar ratio substrate/Ru = 600, c^0^_substrate_ = 0.2 mmol/mL, *T* = 30 °C ^a^calculated as the amount of Ru per weight unit of substrate

**Figure 4:**
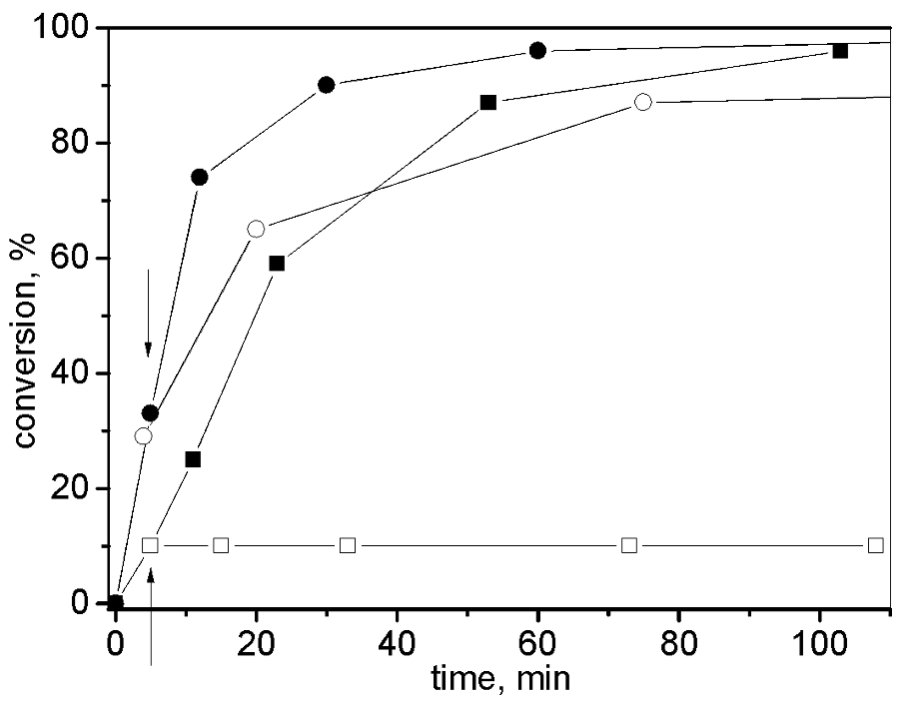
Filtration experiments with **3**/SBA-15. RCM of DEDAM in benzene (circles), 1,7-octadiene in cyclohexane (squares), liquid phase in contact with solid catalyst (filled symbols), liquid phase after filtration (open symbols), arrows indicate time of filtration. Substrate/Ru molar ratio 600, *T* = 30 °C, c^0^_DEDAM_ = 0.22 mol/L, c^0^_1,7-octadiene_ = 0.16 mol/L.

Filtration experiments carried out with **3**/SBA-15 (Figure 4) showed that the catalytic activity was completely bound to the solid phase for the RCM of 1,7-octadiene in cyclohexane. At 5 min after starting the reaction, about ½ of the volume of the liquid phase was separated by filtration and transferred into a new reactor, kept under identical reaction conditions. No increase in conversion was observed in this reactor. For the RCM of DEDAM in benzene, however, a considerable increase of substrate conversion in liquid phase after its separation from solid catalyst indicated that the Ru species released from the solid catalyst were capable of initiating catalytic reactions.

**Figure 5:**
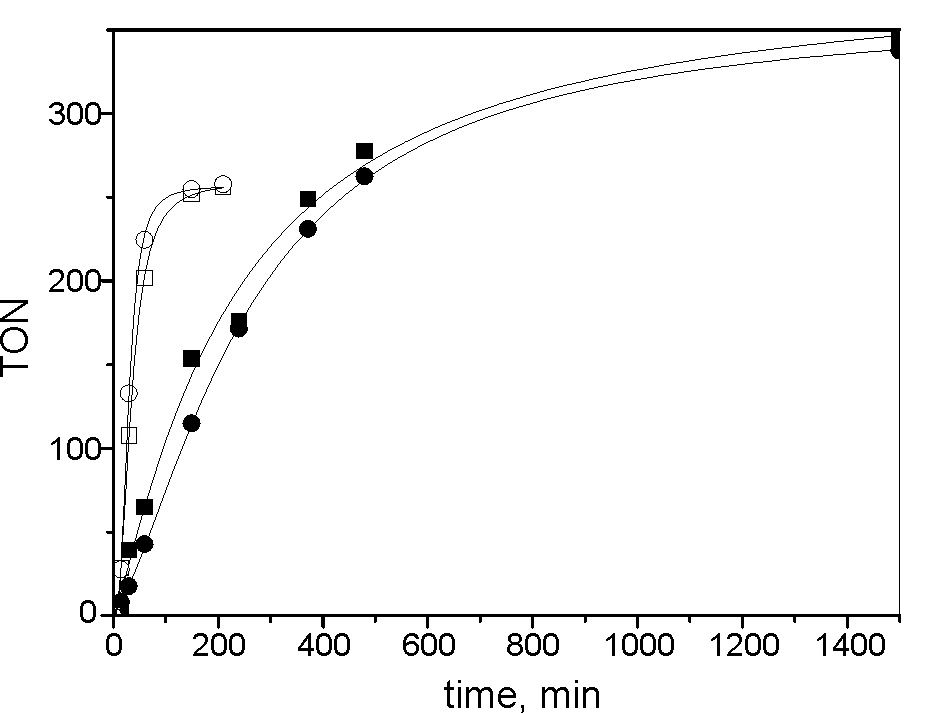
Metathesis of methyl oleate (open symbols) and methyl 10-undecenoate (filled symbols) with **3**/MCM-41 (circles) and **3**/SBA-15 (squares). Molar ratio substrate /Ru = 500, *T* = 30 °C, c^0^_substrate_ = 0.15 mol/L.

Figure 5 shows the activity of **3**/MCM-41 and **3**/SBA-15 in the metathesis of methyl oleate and methyl 10-undecenoate. The reaction proceeded more slowly than in the case of the RCM of DEDAM (TOF at 30 min = 260 h^−1^ for methyl oleate and 35 h^−1^ for methyl 10-undecenoate, both with **3**/MCM-41). The metathesis of methyl oleate reached the equilibrium after 2 h. In the case of methyl 10-undecenoate, the reaction rate was lower than for methyl oleate (because of probable non-productive metathesis [23]) and the final conversion was about 65% (due to the evolution of ethylene into the gas phase). There was no significant difference observed in the conversion curves for reactions with **3**/MCM-41 and **3**/SBA-15. Selectivity near 100% was achieved in all cases.

The ROMP reactions were carried out for cyclooctene with both **3**/MCM-41 and **3**/SBA-15 (cyclooctene/Ru molar ratio = 500, c^0^_cyclooctene_ = 0.6 mol/L, *T* = 30 °C). High molecular weight polymers (*M*_w_ = 250 000, *M*_n_ = 110 000) were obtained in high yields (70% and 64% for **3**/MCM-41 and **3**/SBA-15, respectively) after 3 h.

The catalysts **3**/MCM-41 and **3**/SBA-15 exhibited some features similar to those of Hoveyda–Grubbs catalyst immobilized on silica [20]: (a) Filtration experiments proved complete catalyst heterogeneity only for nonpolar solvents (cyclohexane and hexane, respectively); (b) catalyst leaching in these solvents was extremely low, ensuring more than one order of magnitude lower Ru concentration in products compared to the upper limit permissible in pharmaceutical production (10 ppm [3]); and (c) similar catalyst activity was observed. Although the slightly different reaction conditions applied in [20] do not allow an exact comparison, the initial TOF values achieved in [20] are of the same order as the TOF values obtained in our work. However, in the case of the ROMP of cyclooctene, catalysts **3**/MCM-41 and **3**/SBA-15 led to high molecular weight polymers, whereas in [20] the formation of polymer was not referred.

### Interaction between the Ru complex and the support

**Figure 6:**
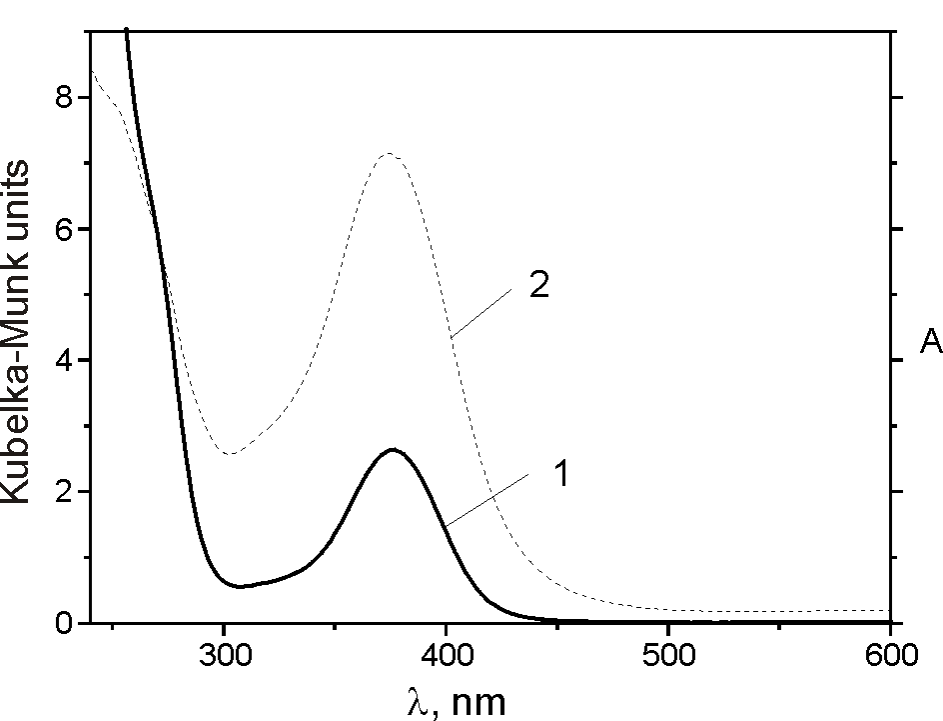
UV–vis spectra of **3**/SBA-15 (curve 2) and of **3** (curve 1) in dichloromethane (c = 0.001 mol/L, *l* = 0.2 cm).

For Hoveyda–Grubbs catalyst immobilized on silica, the authors in [20] proposed a direct chemical interaction between the Ru species and the silanol groups of the surface, instead of a weak physisorption. In order to shed light on the mode of complex attachment to the sieve surface, UV–vis and XPS spectra were measured. In Figure 6, the UV–vis spectra of **3**/SBA-15 and **3** in CH_2_Cl_2_ are compared. The bands at λ = 375 nm and at λ = 600 nm (2 orders of magnitude lower ε, not visible in Figure 6) in the spectrum of **3** reflect the d–d transition of the Ru(II) atoms [24]. Supported catalyst **3**/SBA-15 exhibits the same spectrum suggesting no changes in the coordination sphere of Ru atoms occurred during immobilization of **3** on the sieve.

Assuming non-covalent interactions between the Ru species and the support surface, we attempted to wash out the Ru species from **3**/SBA-15 with THF-*d*_8_ and characterise the eluate by NMR spectroscopy. About 100 mg of **3**/SBA-15 was mixed with 1.5 mL of THF-*d*_8_ and stirred for 2 h at room temperature. The dark green supernatant was then transferred into a NMR tube and the solid phase was washed with THF and dried in vacuo. According to the elemental analysis, 76% of the Ru was removed. In the supernatant, the presence of compound **3** was demonstrated by comparing ^1^H and ^13^C NMR spectra of the supernatant and corresponding spectra of a fresh solution of **3** in THF-*d*_8_***.*** The catalytic activity of the solid phase was then tested in the RCM of DEDAM in cyclohexane (molar ratio DEDAM/Ru = 600, *T* = 30 °C, c^0^_DEDAM_ = 0.2 mol/L). After 1 h, 55% conversion of DEDAM was achieved and 62% after 6 h. Only the RCM products were observed. This indicates that the remaining Ru species after washing was catalytically active; however, these species were rapidly deactivated.

**Figure 7:**
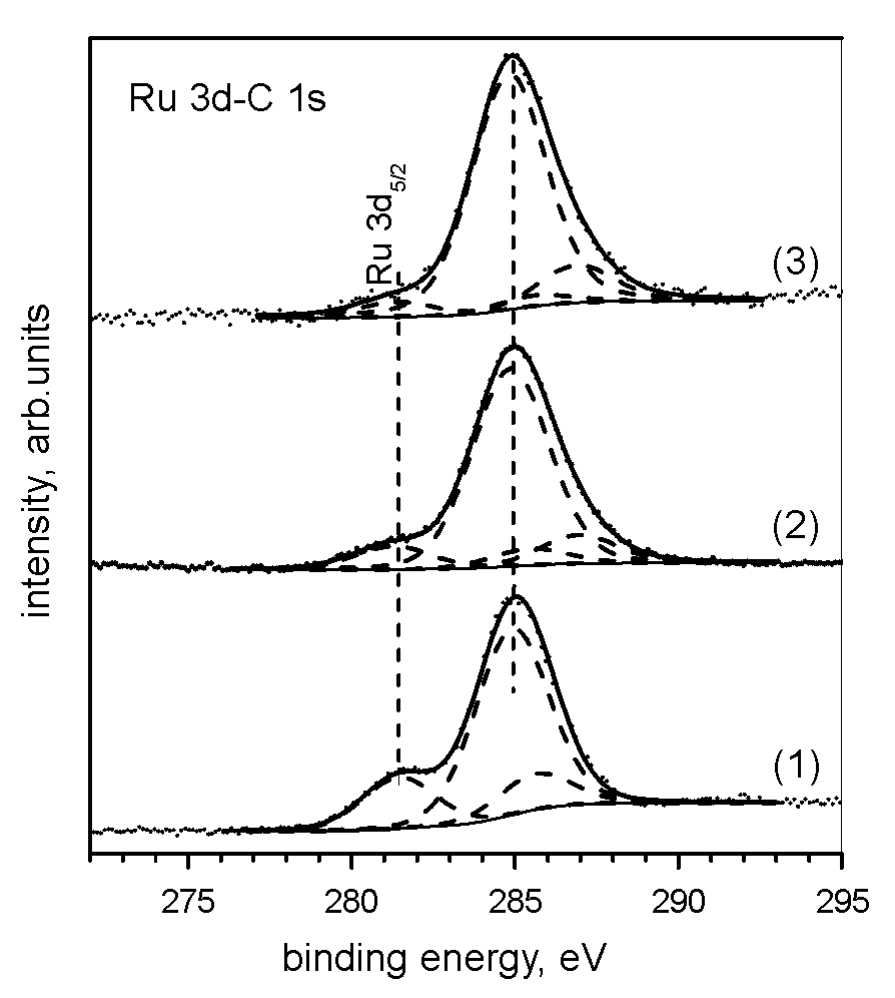
Spectra of the Ru 3d–C 1s photoelectrons for neat compound **3** (1), catalyst sample **3**/SBA-15 (2) and catalyst sample **3**/SBA-15 after leaching in THF (3).

Figure 7 shows high-resolution spectra of the Ru 3d–C 1s photoelectrons of neat compound **3**, catalyst **3**/SBA-15 and the same catalyst after leaching in THF. The measured binding energy of the Ru 3d_5/2_ electrons (281.2 ± 0.2 eV) was identical for all these samples and was in accord with the value 280.95 eV reported in literature [25] for a similar compound. This result indicates that the structure of the Ru complex is not substantially changed by the immobilization on the support surface. This conclusion is corroborated by the results of quantitative analysis. For this catalyst, the atomic concentration ratios Ru/Si = 3.5 × 10^−3^ and Cl/Ru = 2.0 were obtained from integrated intensities of Ru 3d, Si 2p and Cl 2p photoemission lines. For the sample leached by THF, the ratio Ru/Si = 9 × 10^−4^, which is in accord with the amount of Ru removed from the support by leaching as determined from elemental analysis.

The results obtained suggest that the Ru species in **3**/SBA-15 were attached to the sieves by a non-covalent interaction (probably via physical adsorption). The small residual Ru content, which was not possible to remove from **3**/SBA-15 with THF at room temperature, might be due to **3** more firmly attached to the surface (e.g., adsorbed at special sites on the surface, or embedded into micropores, etc.). Because of the low concentration of these species, we have not been able to investigate their bonding to the surface in greater detail.

## Conclusion

The Hoveyda–Grubbs type catalyst **3** was immobilized on mesoporous molecular sieves MCM-41 and SBA-15 by mixing a suspension of **3** and sieves in toluene at room temperature. The immobilization proceeded quickly and almost quantitatively. Heterogeneous catalysts prepared in this way exhibited high activity and 100% selectivity in the RCM of 1,7-octadiene and diethyl diallylmalonate, in the metathesis of methyl oleate and methyl 10-undecenoate, and in the ROMP of cyclooctene. Ru leaching was found to depend on the polarity of substrate and solvent used. The lowest leaching was found for the RCM of 1,7-octadiene in cyclohexane – 0.04% of catalyst Ru content. On the other hand, for the RCM of diethyl diallylmalonate in dichloromethane, leaching reached 14% of initial Ru content in the catalysts.

Results from UV–vis and XPS studies suggested that **3** was attached to the sieve surface by non-covalent interactions. Approximately 76% of the Ru content could be recovered from the sieve as **3** (as shown by NMR) by washing with THF at room temperature (indicating physical adsorption of **3** on the sieve). The residual Ru species on the sieve exhibited catalytic activity in RCM but were rapidly deactivated.

The advantage of these catalysts is their simple preparation, avoiding support modification with special linker molecules. In nonpolar systems, they can function as true efficient heterogeneous catalysts. In the case of polar systems, the possibility of Ru leaching to a significant extent should be taken into account.

## Experimental

### Materials

Toluene was dried overnight over anhydrous Na_2_SO_4_, then distilled with Na and stored over molecular sieves 4 Å. Benzene (Lach-Ner, Czech Republic) was dried over anhydrous Na_2_SO_4_, distilled with P_2_O_5_ and then with NaH in vacuo. Dichloromethane (Lach-Ner) was dried overnight over anhydrous CaCl_2_, then distilled with P_2_O_5_ and stored over molecular sieves 4 Å. Cyclohexane was distilled with P_2_O_5_, then dried with CaH_2_ and stored over molecular sieves 4 Å. Diethyl diallylmalonate (Sigma-Aldrich, purity 98%), cyclooctene (Janssen Chimica, purity 95%), 1,7-octadiene (Fluka, purity ≥ 97.0%), methyl oleate (Research Institute of Inorganic Chemistry, a.s., Czech Republic, purity 94%: methyl palmitate, methyl stearate and methyl linolate as the main impurities) were used as received**.** Methyl 10-undecenoate was prepared from 10-undecenoic acid [26]. The Ru complex **3** was purchased from Zannan Pharma. Ltd., China.

The synthesis of siliceous MCM-41 and SBA-15 was performed according to the procedure described in [27]. Their textural characteristics were determined for MCM-41 and SBA-15, respectively, from nitrogen adsorption isotherms: surface area *S*_BET_ = 972 and 934 m²/g, average pore diameter d = 3.8 and 6.9 nm and volume of pores *V* = 1.14 and 0.96 cm³/g. The particle size (by SEM) ranged from 1 to 3 μm for all supports used.

### Techniques

UV–vis spectra of catalysts were recorded with a Perkin-Elmer Lambda 950 spectrometer. A Spectralon integration sphere was applied to collect diffuse reflectance spectra of powder samples. Spectralon served also as a reference. Catalyst samples were placed in a quartz cuvette under an Ar atmosphere. ^1^H (300 MHz) and ^13^C (75 MHz) NMR spectra were recorded on a Varian Mercury 300 spectrometer in THF-*d*_8_ at 25 °C.

The photoelectron spectra of the samples were measured with an ESCA 310 (Scienta, Sweden) spectrometer equipped with a hemispherical electron analyzer operated in a fixed transmission mode. Monochromatic Al Kα radiation was used for the electron excitation. The samples were spread on aluminium plates and the spectra were recorded at room temperature. The Si 2p, O 1s, Cl 2p, C 1s and Ru 3d photoelectrons were measured. Sample charging was corrected using the C 1s peak at 284.8 eV as internal standard. For the overlapping C 1s and Ru 3d lines, the contributions of individual components were determined by curve fitting. The spectra were curve-fitted after subtraction of the Shirley background [28] using a Gaussian–Lorentzian line shape. The decomposition of the Ru 3d and C 1s profiles was made subject to the constraints of the constant Ru 3d doublet separation (4.17 eV) and the constant Ru 3d_5/2_/Ru 3d_3/2_ intensity ratio equal to the ratio of the corresponding partial photoionization cross sections (1.45) [29]. Quantification of the elemental concentrations was accomplished by correcting photoelectron peak intensities for their cross sections, analyzer transmission function and assuming a homogeneous sample.

A high-resolution gas chromatograph Agilent 6890 with DB-5 column (length: 50 m, inner diameter: 320 μm, stationary phase thickness: 1 μm) was used for reaction product analysis. Nonane was used as an internal standard when required. The Ru content was determined by ICP-MS (by Institute of Analytical Chemistry, ICT, Prague).

### Catalyst preparation

About 1 g of support was transferred into a Schlenk tube and dried for 3 h at 300 °C in vacuo. After drying, the Schlenk tube was filled with argon. Then 10–20 mL of toluene and a calculated amount of **3** was added with stirring at room temperature. After stirring for 30 min, the solid phase turned green and the supernatant became colorless. Then, the supernatant was removed by filtration and the solid catalyst was washed two times with 10 mL toluene. Finally, the catalyst was dried in vacuo at room temperature.

#### Catalytic experiments

Catalytic experiments were performed in Schlenk tubes under an Ar atmosphere in CH_2_Cl_2_ or cyclohexane. In a typical experiment, 1,7-octadiene (225 mg, 2.05 mmol) was added to **3**/SBA-15 (34 mg, 3.4 μmol of Ru) in cyclohexane (10 mL) at 30 °C with stirring. Samples of the reaction mixture (100 μL) were taken at given intervals, quenched with ethyl vinyl ether and analyzed by GC. In the ROMP experiments, the reaction conditions were similar to those for the RCM of 1,7-octadiene, with an initial concentration of cyclooctene of 0.6 mol/L. After 3 h, the reaction mixture was terminated with ethyl vinyl ether, the solid catalyst was separated by centrifugation and the polymer isolated by precipitation in methanol (containing 2,6-di-*tert*-butyl-*p*-cresol as an antioxidant). The polymer yield was determined gravimetrically. The molecular weight was determined by SEC and the values related to the polystyrene standards.
